# Significance of transesophageal contrast echocardiography with the agitated saline test for diagnosing pulmonary arteriovenous malformations

**DOI:** 10.3389/fcvm.2022.975901

**Published:** 2022-09-08

**Authors:** Kyung Hee Lim, Sung Mok Kim, Sung-Ji Park, Eun Kyoung Kim, Sung-A Chang, Sang-Chol Lee, Seung Woo Park, Yeon Hyeon Choe

**Affiliations:** ^1^Division of Cardiology, Department of Internal Medicine, Dong-A University Hospital, Busan, South Korea; ^2^Department of Radiology, Imaging Center, Heart Vascular Stroke Institute, Samsung Medical Center, Sungkyunkwan University School of Medicine, Seoul, South Korea; ^3^Division of Cardiology, Department of Internal Medicine, Imaging Center, Heart Vascular Stroke Institute, Samsung Medical Center, Sungkyunkwan University School of Medicine, Seoul, South Korea

**Keywords:** pulmonary arteriovenous malformation, transesophageal echocardiography, cerebral infarction, shunt, idiopathic

## Abstract

**Objectives:**

This study evaluated the diagnostic value of transesophageal contrast echocardiography (TECE) with the agitated saline test for diagnosing pulmonary arteriovenous malformations (PAVMs) in comparison with chest CT or pulmonary angiography.

**Background:**

Although transthoracic contrast echocardiography (TTCE) is the recommended screening test for diagnosing PAVMs, it has low specificity and positive predictive value. TECE is expected to offer improved sensitivity and specificity compared with TTCE, but no studies have reported the diagnostic accuracy and clinical significance of TECE in detecting PAVMs.

**Methods and results:**

In total, 1,809 patients underwent TECE with the agitated saline test to evaluate symptoms of a suspected right to left shunt. Patients with hereditary hemorrhagic telangiectasia (HHT) were excluded. A total of 387 patients showed transpulmonary bubble passage, indicating a PAVM. Among them, 182 patients had additional chest CT or pulmonary angiography. Those patients were divided into two groups according to the presence of a PAVM in the radiologic imaging. A total of 18 patients (9.8%) were confirmed for the PAVM group. Only 13 patients required embolization for their PAVMs. The TECE with saline test results were divided into four grades according to the number of bubbles: grade 1 (*n* = 91), grade 2 (*n* = 47), grade 3 (*n* = 35), and grade 4 (*n* = 9). None of the patients in the PAVM group had grade 1 shunts in their TECE results. The positive predictive values for the presence of a PAVM according to the TECE grade scale were 10.6% for grade 2, 22.8% for grade 3, and 55.6% for grade 4.

**Conclusion:**

TECE with a grade scale is a useful method for initially diagnosing PAVMs in non-HHT patients with a suspected right to left shunt. The findings of this study also suggest that patients with a small grade (<10 bubbles) shunt in their TECE findings should be spared unnecessary radiation exposure from CT scans or pulmonary angiography.

## Introduction

Pulmonary arteriovenous malformations (PAVMs) are pulmonary vascular anomalies that enable capillary-free communication between the pulmonary artery and pulmonary vein (1). PAVMs are mostly hereditary, and approximately 90% of them are associated with hereditary hemorrhagic telangiectasia (HHT) (2–4). Although PAVMs are rare, untreated PAVMs contribute to complications, such as paradoxical arterial embolization, stroke, cerebral abscess, hypoxemia, migraine, and hemorrhage (5–7). Previous studies have already established effective screening tests for PAVM diagnosis in asymptomatic HHT patients. Transthoracic contrast echocardiography (TTCE) has high sensitivity (93–100%) for detecting PAVMs (3, 8–10). Because complications associated with PAVMs also occur in even asymptomatic patients without HHT and because PAVMs can be effectively treated by percutaneous transcatheter embolization (11), the diagnosis of PAVMs that form an intrapulmonary right-to-left shunt in patients without HHT is important.

In patients suspected to have PAVMs, TTCE with the agitated saline test is recommended as the initial screening modality instead of chest CT or diagnostic pulmonary angiography (1, 12). In TTCE with the agitated saline test, the delayed appearance of bubbles in the left atrium (LA), more than three to five cardiac cycles after initial opacification of the right atrium, is considered to indicate an extracardiac shunt, including PAVMs (13). However, the presence of PAVMs is not confirmed by the introduction of bubbles from the pulmonary vein into the LA in TTCE, which has relatively low specificity for radiographically detectable PAVMs (32–82%) (3, 8, 9). A false positive indication of an extracardiac shunt in TTCE can result in unnecessary radiation exposure from chest CT or pulmonary angiography. Transesophageal contrast echocardiography (TECE) with agitated saline might be a more specific screening diagnostic test for detecting a right to left shunt, though it is a more invasive procedure than TTCE (14). Some studies have already tested TECE with the agitated saline test for the right to left shunt detection (15–17), but no studies have reported the diagnostic accuracy or clinical significance of using TECE with the agitated saline test to detect PAVMs. We hypothesized that TECE with the agitated saline test would be a useful initial screening tool for identifying PAVMs in patients with a suspected right to left shunt because it visualizes transpulmonary bubble passage.

## Materials and methods

### Study population

**FIGURE 1:**
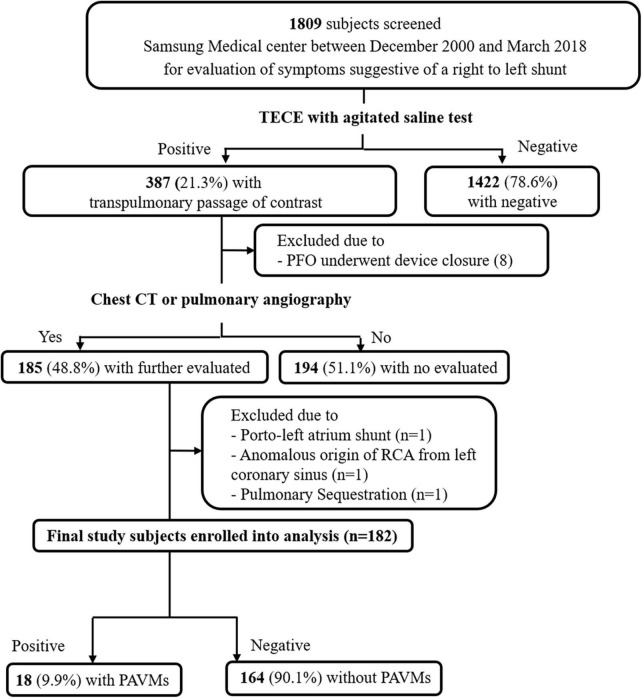
A schematic diagram of study patient selection. TECE, transesophageal contrast echocardiography; PFO, patent foramen ovale; CT, computed tomography; RCA, right coronary artery; PAVM, pulmonary arteriovenous malformation.

We retrospectively investigated 1,809 subjects who underwent TECE with the agitated saline test at Samsung Medical Center between December 2000 and March 2018 for evaluation of symptoms suggestive of a right to left shunt. The Curacao diagnostic criteria were used to distinguish and exclude patients with HHT. Those criteria are recurrent epistaxis, telangiectasia, arteriovenous malformations, and family history of HHT, and an HHT diagnosis is considered definite if three of those conditions are met. None of the patients analyzed in this study were found to have definite HHT. Among the 387 subjects who had the transpulmonary passage of contrast in their TECE results, which suggested the presence of a PAVM, 185 subjects underwent a further imaging study such as chest CT or pulmonary angiography at their clinician’s discretion. The time interval between echocardiography and the further imaging study was less than 120 days in all patients. Patients with an extracardiac shunt (1 porto-left atrium shunt; 1 anomalous origin of the right coronary artery from the left coronary sinus, 1 pulmonary sequestration) and those with patent foramen ovale requiring device closure (*n* = 8) were excluded. Thus, 182 patients were enrolled in this study and divided into two groups: 18 patients with PAVMs and 164 without PAVMs (Figure 1). The following clinical data were collected: age, sex, underlying disease, smoking history, routine blood lab, transthoracic echocardiography, transesophageal echocardiography, and PAVM embolization.

### Transthoracic echocardiography and transesophageal echocardiography

**FIGURE 2:**
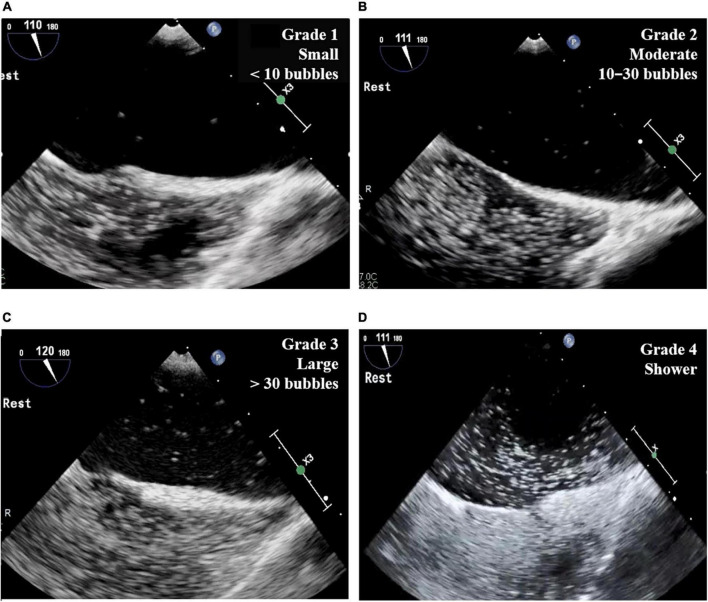
Grade of TECE with agitated saline test. **(A)** Grade 1 (small): fewer than 10 bubbles in LA; **(B)** grade 2 (moderate): 10–30 bubbles in LA; **(C)** grade 3 (large): more than 30 bubbles in LA; **(D)** grade 4 (shower): extensive contrast in LA. TECE, transesophageal contrast echocardiography; LA, left atrium.

The two-dimensional and Doppler echocardiographic examinations were performed in a standard manner (18, 19) using commercially available echocardiographic devices. Standard TECE with the agitated saline test was performed in study patients by an experienced echocardiography specialist with the support of a sonographer. Three-way stopcocks with 10 ml syringes connected to both ends were placed in the patient’s forearm veins. The saline contrast was composed of 8–9 ml of normal saline agitated with 0.5 ml of air and a small amount of the patient’s blood. In the first study with each patient, the agitated saline contrast test was performed during rest in the bicaval view (110° view). The second test was performed with the Valsalva maneuver or coughing in the bicaval view or a 45° view. Then, the saline contrast test was performed without the Valsalva maneuver in the right and left pulmonary vein select view. In each test, the number of cardiac cycles was measured between the full appearance of air bubbles in the right atrium and their appearance in the LA. The number of bubbles and the location of the shunt were also recorded. The number of bubbles in the LA was graded according to a 4-grade system: grade 1, a small amount of contrast or fewer than 10 bubbles; grade 2, a moderate amount of contrast or 10–30 bubbles; grade 3, more than 30 bubbles; grade 4, a shower with extensive contrast in the LA (Figure 2). The TECE classification of shunt size was based on the grading system for patent foramen ovale (20), which was modified slightly to facilitate the evaluation of shunt size. Our grading system also differentiates the shower grade in accordance with the TTCE grading system, which is another screening test for diagnosing PAVMs (9).

### Computed tomography, pulmonary angiography, and embolization

Contrast-enhanced chest CT images with 1-mm reconstructions in the axial and 2-mm reconstructions in the coronal planes were used for analysis. The identification of at least one feeding artery and vein is mandatory for establishing a PAVM diagnosis on CT images (21) and pulmonary angiography. The diameter of the feeding arteries was measured as close as possible to the nidus of the PAVM. Catheter angiography is not performed routinely for diagnosing PAVMs, but it has been generally performed during embolization procedures. We confirmed PAVMs using the non-selective left and right pulmonary arteriography, which was performed through a 5-Fr Pigtail catheter using the right femoral venous approach. Because all patients had symptoms suggestive of a right to left shunt, embolization was used actively in the PAVM group as much as possible. However, for small PAVMs whose nidus was not visible in the radiologic imaging test, meaning that the connection between the artery and vein was only suspected, embolization was not performed. PAVMs that were not treated with embolization all had a feeding artery that was measured to be less than 1 mm. The Nidus and feeding artery size of a PAVM are described in Supplementary Table 1.

### Statistical analysis

Continuous variables are presented as medians and interquartile ranges. The Mann-Whitney test was used to compare between two groups. Categorical variables are presented as absolute numbers (n) and percentages (%), and differences were analyzed using the Pearson chi-squared test. A logistic regression analysis was performed to determine the association between the grade of a shunt in the TECE findings and the presence of PAVMs on CT or pulmonary angiography. The positive predictive value (PPV) of each TECE with agitated saline test grade was calculated using the chest CT or pulmonary angiography result as a reference. All tests were two-tailed, and *p*-values < 0.05 were considered significant. Statistical analysis was performed with SPSS version 23 (IBM Corp., Armonk, NY, United States).

## Results

**FIGURE 3:**
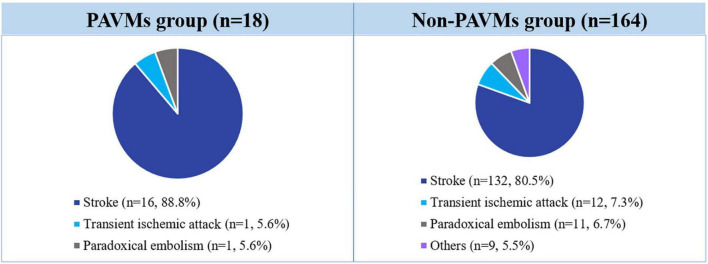
Incidence of symptoms associated with PAVM requiring TECE with the agitated saline test. TECE, transesophageal contrast echocardiography; PAVM, pulmonary arteriovenous malformation.

Of the 1,809 subjects who underwent TECE with the agitated saline test who were reviewed in this study, 387 (21.3%) patients had confirmed the presence of transpulmonary bubble passage in their TECE findings, which was considered to indicate the presence of a PAVM. Only the 182 patients who underwent a further image study (chest CT or pulmonary angiography) were finally included in this study and divided into the PAVM group (*n* = 18) and the non-PAVM group (*n* = 164). All patients had symptoms suggestive of right to left shunts, such as a PAVM. The main symptomatic reasons for performing TECE with the agitated saline test were stroke (*n* = 148, 81.3%), transient ischemic attack (*n* = 13, 7.1%), paradoxical arterial embolism (*n* = 12, 6.6%), and other, including migraine and hypoxia (*n* = 9, 4.9%). Further information is provided in Figure 3.

The baseline characteristics and laboratory findings are shown in Table 1. The PAVM group had more female patients (*p* = 0.001), and there were differences in the proportion of concomitant disease between the PAVM and non-PAVM groups. Malignancy was more frequent in the non-PAVM group [PAVM vs. non-PAVM; 0 (0%) vs. 30 (18.3%), *p* = 0.047], and the non-PAVM group also had a higher prevalence of dyslipidemia [2 (11.1%) vs. 55 (33.5%), *p* = 0.062], and history of smoking [2 (11.1%) vs. 52 (31.7%), *p* = 0.069]. The mean blood creatinine level was lower in the PAVM group than in the non-PAVM group, but all those values were within the normal range. None of the other baseline characteristics differed statistically between the two groups.

**TABLE 1:** Clinical characteristics and laboratory findings.

Characteristic	PAVM group (*n* = 18)	Non-PAVM group (*n* = 164)	*p*
Male sex, *n* (%)	3 (16.7%)	96 (58.5%)	0.001*
Age	45 [41–48]	50 [36–69]	0.072
Diabetes mellitus	0 (0%)	17 (10.4%)	0.226
Dyslipidemia	2 (11.1%)	55 (33.5%)	0.062
Liver cirrhosis	0 (0%)	4 (2.4%)	1.000
Chronic kidney disease	1 (5.6%)	5 (3%)	0.470
Infection/inflammation	0 (0%)	13 (7.9%)	0.369
Malignancy	0 (0%)	30 (18.3%)	0.047*
SPN (lung)	0 (0%)	18 (11%)	0.223
Smoking history	2 (11.1%)	52 (31.7%)	0.069
Hemoglobin	13 [12.3–13.9]	13.6 [12.4–14.9]	0.170
Creatinine	0.65 [0.58–0.69]	0.79 [0.68–0.93]	0.002*

The p-value denotes statistical significance when comparing the PAVM and non-PAVM groups.

*P < 0.05 by Mann-Whitney test or χ2-test. Data are listed as numbers (percentage of the group) or median and interquartile ranges.

PAVM, pulmonary arteriovenous malformation; SPN, solitary pulmonary nodule.

Transthoracic echocardiography was performed to evaluate cardiac function and chamber size. Left ventricle (LV) systolic function and size, LV diastolic function (assessed by septal e’), average E/e’, LA volume index, and peak tricuspid regurgitation velocity did not differ significantly between the two groups. However, median peak e’ wave velocity was greater in the PAVM group than in the non-PAVM group [0.11 (0.09–0.11) vs. 0.09 (0.07–0.11), *p* = 0.022]. Further transthoracic echocardiographic parameters are summarized in Table 2.

**TABLE 2:** Transthoracic echocardiography parameters.

Characteristic	PAVM group (*n* = 18)	Non-PAVM group (*n* = 164)	*p*
LV end-diastolic dimension, mm	47.0 [45.9–48.0]	48.0 [45.0–51.0]	0.277
LV ejection fraction,%	64.5 [59.8–68.4]	64.0 [59.0–68.0]	0.986
Mitral E velocity, cm/s	0.74 [0.65–0.87]	0.71 [0.56–0.82]	0.313
Mitral A velocity, cm/s	0.54 [0.46–0.59]	0.60 [0.49–0.76]	0.061
E/A ratio	1.47 [1.20–1.68]	1.09 [0.84–1.53]	0.029*
Medial e’ velocity, cm/s	0.11 [0.09–0.11]	0.09 [0.07–0.11]	0.022*
Medial E/e’	7.3 [6.4–8.2]	8.5 [6.5–9.8]	0.068
LA volume index, mL/m^2^	27.6 [22.6–34.5]	29.9 [25.8–35.1]	0.170
**Diastolic function**			0.322
Normal	16 (88.9%)	121 (73.7%)	
Grade I dysfunction	0	19 (11.6%)	
Grade II dysfunction	0	6 (3.7%)	
Grade III dysfunction	0	2 (1.2%)	
Indeterminate	2 (11.1%)	16 (9.8%)	

The p-value denotes statistical significance when comparing the PAVM and non-PAVM groups.

*P < 0.05 by Mann-Whitney test or χ^2^-test. Data are listed as numbers (percentage of the group) or median and interquartile ranges.

PAVM, pulmonary arteriovenous malformation; LV, left ventricular; LA, left atrium.

TTCE with the agitated saline test is described in Table 3. The TECE parameters assessed between the PAVM and non-PAVM groups were the number of cardiac onset cycles in the LA, the location of the shunt, and the number of bubbles according to the grade scale (without the Valsalva maneuver). The number of cardiac cycles for bubbles to appear in the LA after fully filling the right atrium was lower in the PAVM group than in the non-PAVM group [3 (2–5) vs. 6 (4–7), *p* < 0.001]. All but 1 patient with a positive transpulmonary passage of bubbles had confirmed origin in the pulmonary vein. The most common origin was the left pulmonary vein in both groups [8 (44.4%) vs. 122 (74.8%)], but the proportion of pulmonary vein origin differed slightly between the groups (*p* = 0.024).

**TABLE 3:** Transesophageal contrast echocardiography with agitated saline test parameters.

Characteristic	PAVM group (*n* = 18)	Non-PAVM group (*n* = 164)	*p*
Number of cardiac onset cycles	3 [2–5]	6 [4–7]	<0.001*
Origin			0.024*
Right pulmonary vein	5 (27.8%)	22 (13.5%)	
Left pulmonary vein	8 (44.4%)	122 (74.8%)	
Both pulmonary veins	5 (27.8%)	19 (11.7%)	
Grade scale			<0.001*
Small (grade 1, < 10)	0 (0%)	91 (55.5%)	
Moderate (grade 2, 10–30)	5 (27.8%)	42 (25.6%)	
Large (grade 3, > 30)	8 (44.4%)	27 (16.5%)	
Shower (grade 4)	5 (27.8%)	4 (2.4%)	

The p-value denotes statistical significance when comparing the PAVM and non-PAVM groups.

*P < 0.05 by Mann-Whitney test or χ^2^-test. Data are listed as numbers (percentage of the group) or median and interquartile ranges.

PAVM, pulmonary arteriovenous malformation.

We also analyzed the TECE with an agitated saline grade scale according to the presence of PAVMs in the CT or pulmonary angiography results. The TECE with agitated saline test results indicated that the PAVM group had a higher frequency of high grade transpulmonary bubble passage than the non-PAVM group (*p* < 0.001). None of the patients in the PAVM group had small grade shunts, whereas the majority of the non-PAVM group had small grade shunts [*N* = 91 (55.5%)]. Further information is provided in Table 3.

Moreover, the TECE with agitated saline test grade scale was further evaluated to determine whether those findings could predict whether PAVMs would be found in CT or pulmonary angiography and whether patients would need embolization. The relationships among the TECE grade scale, presence of PAVMs in CT or pulmonary angiography, and presence of PAVMs that required embolization are shown in Table 4. The TECE grade scale and presence of PAVMs in chest CT or pulmonary angiography showed a significant positive correlation in our study patients (*p* < 0.001). Also, there was a significant association between increases in the TECE shunt grade scale and PAVMs requiring embolization, as expected (*p* < 0.001). Eighteen patients (100%) in the PAVM group had their PAVM locations evaluated through TECE using the pulmonary vein select view. Table 5 shows the comparison of PAVM locations assessed by TECE, CT, and pulmonary angiography. All the recorded characteristics of the PAVM group are described in Supplementary Table 1. The TECE results of five patients (27.8%) confirmed the presence of a PAVM in the right lung, and all of them underwent embolization of PAVMs in the right lung. The TECE results of eight patients (44.4%) indicated PAVMs in the left lung, and half of them received embolization for those PAVMs. Only one patient was found to have PAVMs in both pulmonary veins in their radiologic imaging study results. Because of the small size or obscure nidus of their PVAMs, embolization was not performed in five patients in the PAVM group.

**TABLE 4:** Relationship between the TECE grade scale and the presence of PAVMs in CT or pulmonary angiography and PAVMs that required embolization.

TECE grade scale	Number	Presence of PAVMs in	*p*	Requiring	*p*
		CT or pulmonary angiography, *n* (%)		embolization, *n* (%)	
			<0.001*		<0.001*
Small (<10)	91	0 (0%)		0 (0%)	
Moderate (10–30)	47	5 (10.6%)		2 (4.3%)	
Large (>30)	35	8 (22.8%)		6 (17.1%)	
Shower	9	5 (55.6%)		5 (55.6%)	

*P < 0.05 by chi-square test for trend. Data are listed as numbers (percentage of the group).

PAVMs, pulmonary arteriovenous malformations; TECE; transesophageal contrast echocardiography; CT, computed tomography.

**TABLE 5:** Comparison of PAVM locations assessed by TECE, a radiologic imaging study (chest CT or pulmonary angiography), and embolization.

TECE	Number	Chest CT or pulmonary angiography	Number	Embolization	Number
Right pulmonary vein	5	Right	5 (100%)	Right	5 (100%)
Left pulmonary vein	8	Left	6 (75%)	Left	4 (50%)
		Right	1 (12.5%)		
		Both	1 (12.5%)		
Both pulmonary	5	Left	1 (20%)	Left	1 (20%)
veins		Right	3 (60%)	Right	3 (60%)
		Both	1 (20%)		

PAVMs, pulmonary arteriovenous malformations; TECE, transesophageal contrast echocardiography; CT, computed tomography.

### Interobserver variability

Of the 182 study patients, 10 patients were randomly selected to evaluate the interobserver variability in the TECE grades assigned by two senior sonographers. In the interobserver variability analysis, the Pearson correlation coefficients were greater than 1.0 (Kappa 1.0).

## Discussion

This single-center, retrospective study analyzed patients who underwent both TECE with the agitated saline test and a radiologic imaging study (chest CT or pulmonary angiography) to evaluate symptoms of a suspected right to left shunt. HHT patients were excluded. Although only 182 patients were finally enrolled, 1,809 cases of TECE with the agitated saline test were reviewed. This study reports the predictive value of TECE with the agitated saline test compared with chest CT or pulmonary angiography for diagnosing PAVMs. The major findings of this study are as follows: (1) Among patients who received TECE with the agitated saline test to diagnose a suspected right to left shunt, a considerable number of patients (21.3%) had positive TECE findings. (2) The non-PAVM group had a higher incidence of malignancy, dyslipidemia, and history of smoking than the PAVM group. (3) The PPV of TECE for the presence of PAVMs was 9.8% in non-HHT patients using the chest CT or pulmonary angiography result as a reference, and the TECE grade scale increased the PPV of the inspection. (4) None of the patients with a small shunt (< 10 bubbles) on their TECE findings showed a PAVM on chest CT or pulmonary angiography.

TTCE with the agitated saline test has been widely used as an initial screening technique for diagnosing PAVMs (1). Several studies have examined the performance of TTCE with the agitated saline test for diagnosing PAVMs and found that it had high sensitivity and negative predictive value (3, 6–9). However, most studies compared the diagnostic value of TTCE with chest CT for finding PAVMs in HHT patients (6–9, 13, 22, 23). Also, it is often difficult to differentiate between an intra-cardiac shunt and an extra-cardiac shunt when using TTCE in clinical practice, which results in low specificity and PPV. TTCE is not effective enough as an imaging modality to prevent unnecessary radiation exposure through chest CT or pulmonary angiography when diagnosing PAVMs. TECE with the agitated saline test, which provides more accurate images, can be used as a screening test to diagnose the presence of PAVMs. To our knowledge, no previous studies have examined TECE with the agitated saline test for diagnosing PAVMs or considered the relationship between a TECE grade scale and the presence of PAVMs on CT or pulmonary angiography findings.

We investigated the predictive value of TECE with the saline shunt test for identifying PAVMs in non-HHT patients who had symptoms associated with PAVMs, such as stroke, transient ischemic attack, or paroxysmal arterial embolism. We further analyzed TECE using the number of bubbles (grade scale), cardiac cycles, and shunt origin. Our findings suggest that TECE with a shunt test could safely be used as a screening test for PAVMs in non-HHT patients.

In this study, 387 (21.3%) of the 1,809 patients who received TECE with the agitated saline test showed positive findings. Of the 182 patients who underwent both TECE and an additional radiologic imaging study, 18 showed radiologic images compatible with PAVMs. Because TTCE detects PAVMs at a microscopic level (7, 24), which does not require embolization and is insignificant, it has a lower PPV than chest CT (6, 9). However, we also found that TECE (any grade) had low PPV (9.8%) even lower than that found in previous TTCE studies (6–9, 13, 22, 23). There are several possible explanations for this result. The previously reported TTCE studies were performed in patients with HHT, who have a PAVM prevalence between 30 and 50% (3, 25). The prevalence of PAVMs in the general population remains uncertain, but it has been estimated at approximately < 0.001% (26, 27). This result might explain why the PPV of this study is lower than in previous studies. Moreover, the lower the TECE grade scales, the more often they are microscopic level PAVMS that are undetectable on radiologic. Several studies about the diagnostic value of TTCE in PAVMs published that the lower TTCE grade scale was associated with a lower prevalence of radiologic detectable PAVM (9, 10). Since this study included relatively many patients with a small grade of TECE, low PPV was reported.

To improve the diagnostic value of TTCE for identifying PAVMs, several recent articles have reported an analysis system that uses grades and the onset of the cardiac cycle (6, 9, 22, 23). The number of bubbles in the LA is usually graded according to the grading system proposed by Barzilai et al. (28) and followed by Zukotynski et al. (29). An additional analysis is performed to prevent unnecessary radiation exposure. However, a grading system with more specific criteria is required to evaluate PAVMs in TECE, which shows high-resolution images, because of the relative non-objectivity of the previous system. We used a 4-grade scale according to the number of bubbles visible in the LA. We found that none of the patients with TECE grade 1 [*N* = 91 (50%)] showed PAVMs on chest CT or pulmonary angiography. Comparing these results with the TTCE grades used by other authors, grade 1 in TTCE with a cut-off of 20 microbubbles was present in 35% of the patients in the study of Parra et al. (9) and 32% of the patients studied by Gazzaniga et al. (8). The PPV of different TECE grades for the presence of PAVMs on chest CT or pulmonary angiography was 10.6% for grade 2, 22.8% for grade 3, and 55.6% for grade 4. Also, among patients with TECE grades 2, 3, and 4 in this study, 4.3, 17.1, and 55.6%, respectively, had PAVMs requiring embolization. The relationships between the TTCE grades and the size of the afferent artery on chest CT were described in a previous study (9).

The present study has several limitations. First, among 1,809 subjects who underwent TECE, only half received both TECE and a further radiologic imaging study. One hundred ninety-four subjects did not undergo chest CT or pulmonary angiography based on the charge physician’s judgment. Our results might thus have been affected by selection bias. Second, because this study included only non-HHT patients with a suspected right to left shunt, only a few patients were finally diagnosed with a PAVM. Last, the grade scale for the TECE findings is a semi-quantitative method that can lead to interobserver variability.

## Conclusion

This is the first study to report the use of TECE with the agitated saline test for diagnosing PAVMs. TECE with the agitated saline test is a reasonable screening test for identifying PAVMs in non-HHT patients with a suspected right to left shunt. TECE with a grade scale helps select patients with PAVMs that can be confirmed in chest CT or pulmonary angiography and require embolization. The grading scale could also help to reduce unnecessary radiation exposure from CT scans or pulmonary angiography in patients with small grade (< 10 bubbles) shunt in their TECE findings.

## Data availability statement

The raw data supporting the conclusions of this article will be made available by the authors, without undue reservation.

## Ethics statement

The studies involving human participants were reviewed and approved by the Institutional Review Board of Samsung Medical Center (IRB No. SMC 2021-11-120-001). Written informed consent for participation was not required for this study in accordance with the national legislation and the institutional requirements.

## Author contributions

KHL, SMK, SJP, EKK, SAC, SCL, SWP, and YHC participated in the conception, design, analysis, interpretation of data, and drafting of the manuscript. All authors have approved it for publication.

## Abbreviations

CT: Computed tomography
HHT: Hereditary hemorrhagic telangiectasia
LA: Left atrium
LV: Left ventricle
PAVM: Pulmonary arteriovenous malformation
PPV: Positive predictive value
TECE: Transesophageal contrast echocardiography
TTCE: Transthoracic contrast echocardiography.
