# Supplementary material for: Significance of transesophageal contrast echocardiography with the agitated saline test for diagnosing pulmonary arteriovenous malformations
Source: Front Cardiovasc Med. 2022 Sep 8;9:975901. doi: 10.3389/fcvm.2022.975901 (PMC9497879; doi:10.3389/fcvm.2022.975901)
Supplement: Supplementary file 1 [file Table_1.DOCX]

Supplementary 1. PAVM group status and management

| Patient number | Symptoms | TECE^b^ grade | TECE onset cycle | TECE location | Type | CT or PA location | PAVM  nidus /feeding artery (mm) | Embolization  (Yes/No, location) | Reason for non-execution of embolization |
| --- | --- | --- | --- | --- | --- | --- | --- | --- | --- |
| #1 | Chronic neurologic deficit | 2 | 6 | Both | Single | Right | 6.0/3.0 | Yes, right |  |
| #2 | Stroke | 2 | 6 | Left | Single | Left | None/0.7 | No | Small PAVM^a^ (no definite nidus) |
| #3 | Stroke | 2 | 5 | Left | Single | Right | None/0.9 | No | Small PAVM (no definite nidus) |
| #4 | Stroke | 2 | 5 | Left | Single | Left | None/0.8 | No | Small PAVM (no definite nidus) |
| #5 | Stroke | 2 | 3 | Right | Single | Right | 13.0/3.8 | Yes, right |  |
| #6 | Paroxysmal embolism | 3 | 2 | Both | Single | Left | 5.0/3.2 | Yes, left |  |
| #7 | Transient ischemic attack | 3 | 6 | Both | Multiple | Both | 1: none/0.7,  2: none/0.8,  3: none/0.7, | No | Small PAVM |
| #8 | Stroke | 3 | 3 | Both | Single | Right | 6.3/2.6 | Yes, right |  |
| #9 | Stroke | 3 | 5 | Both | Single | Right | 10.5/2.9 | Yes, right |  |
| #10 | Stoke | 3 | 5 | Left | Multiple | Both | N.A. | No | Small PAVM (no definite nidus) |
| #11 | Stoke | 3 | 3 | Left | Single | Left | 40/1.6 | Yes, left |  |
| #12 | Stoke | 3 | 1 | Left | Single | Left | 6.2/1.8 | Yes, left |  |
| #13 | Stoke | 3 | 3 | Left | Single | Left | 7.0/2.2 | Yes, left |  |
| #14 | Stoke | 4 | 2 | Left | Single | Left | 10.0/4.2 | Yes, left |  |
| #15 | Stroke | 4 | 1 | Right | Multiple | Right | 14.0/3.1 | Yes, right |  |
| #16 | Stroke | 4 | 2 | Right | Sigle | Right | 9.4/3.5 | Yes, right |  |
| #17 | Stroke | 4 | 2 | Right | Multiple | Right | 1: 8.7/2.3,  2: 3.9/2.1,  3: 7.4/5.2,  4: 5.2/1.7, | Yes, right |  |
| #18 | Stroke | 4 | 2 | Right | Single | Right | 5.2/1.9 | Yes, right |  |
